# Preserving malignant provenance in single-cell cancer atlases: lessons from Ewing sarcoma plasticity

**DOI:** 10.1016/j.neo.2026.101392

**Published:** 2026-11-06

**Authors:** Ge Zhao, Zhijie Hu, Baicheng Yang, Wei Shi

**Affiliations:** a Department of Orthopedics, The First Affiliated Hospital of Hebei North University, Zhangjiakou 075000, China; b Department of Orthopedic Surgery, National Cancer Center/National Clinical Research Center for Cancer, Cancer Hospital, Chinese Academy of Medical Sciences and Peking Union Medical College, Beijing 100021, China

**Keywords:** Cancer single-cell atlases, Cell-state plasticity, Ewing sarcoma, Malignant provenance, Tumor microenvironment, Annotation leakage

## Abstract

Single-cell atlases increasingly organize tumors according to recurrent transcriptional states, but state-based integration can weaken or obscure the molecular evidence that distinguishes malignant from non-malignant provenance. Ewing sarcoma provides a mechanistically tractable example because FET::ETS fusions anchor malignant provenance while EWSR1::FLI1 regulatory output spans proliferative, migratory, mesenchymal and matrix-producing states, including cancer-associated fibroblast-like malignant populations. We propose malignant-to-stromal annotation leakage as a testable post-identification failure mode in which provenance-defining evidence is weakened or discarded during reference mapping, annotation or atlas reuse, allowing transcriptional similarity to become a surrogate for cellular identity. Such misassignment can distort estimates of stromal abundance, cell–cell communication, prognostic signatures and therapeutic target attribution. We therefore propose a provenance-aware architecture that keeps malignant provenance, oncogenic regulatory activity, phenotypic state and spatial or clinical context separately traceable while retaining uncertainty. We further outline an evidence hierarchy and a falsifiable benchmarking strategy based on orthogonal molecular evidence, supported where possible by spatial and pathological validation. Although Ewing sarcoma provides the principal exemplar, the framework is designed for testing in other tumors in which plastic malignant states converge transcriptionally with non-malignant compartments.

## Highlights

• Malignant provenance can persist despite stromal-like transcriptional states.
• Ewing sarcoma exposes a testable risk of malignant-to-stromal annotation leakage.
• Provenance, oncogenic activity, state and context should remain separable.
• Provenance-aware annotation may improve biomarker and target attribution.

## Cell-state plasticity challenges provenance assignment in cancer single-cell atlases

Single-cell and spatial atlases increasingly organize tumors around recurrent malignant and microenvironmental states. Pan-cancer resources such as TabulaTIME and the Curated Cancer Cell Atlas integrate millions of cells, enabling immune and stromal programs to be compared across diseases in shared latent spaces [1,2]. This common vocabulary is valuable, but it creates a specific interpretive challenge when plastic malignant cells converge transcriptionally with normal or stromal compartments. In that setting, current phenotype alone cannot establish whether a cell belongs to a malignant clone.

Integration methods must balance removal of technical and study-specific variation against preservation of biological differences [3]. We distinguish four related but non-equivalent layers: malignant provenance identifies tumor-clonal versus non-malignant origin; oncogenic activity measures driver-regulatory output; phenotypic state describes current behavior; and context records sampling site, treatment and spatial or clinical pressures. Here, malignant provenance denotes membership in the tumor clone, supported where possible by independent clonal or genomic evidence; it is distinct from both developmental cell of origin and current phenotypic state. State similarity alone cannot establish provenance, and driver abundance alone cannot substitute for regulatory output.

This distinction matters because atlas state vocabularies are intentionally transferable. A fibroblast-like, hypoxic or invasive program may be biologically comparable across specimens even when it occurs in cells with different clonal origins. The error arises only when comparability of state is converted into equivalence of identity. A provenance-aware atlas should therefore permit the same state label to recur across malignant and non-malignant compartments while retaining the evidence that distinguishes them.

Modern atlases often preserve source labels, tumor-specific markers and inferred copy-number profiles. Cross-tumor integration remains valuable, provided that patient-specific fusion, mutation, copy-number and source-study evidence remains linked to each cell and remains auditable through harmonization and reference reuse. Recent work has addressed how malignant cells can be identified using markers, copy-number alterations, mutations and fusions [4]. Our concern is different: this is a post-identification problem. Once malignant provenance has been established, the evidence supporting that call should remain attached during integration, reference mapping and atlas reuse. If provenance-defining evidence is discarded or weakened, transcriptional state similarity may silently become a surrogate for cellular identity.

This Opinion Article synthesizes published experimental and single-cell evidence; it reports neither a new dataset nor an original reanalysis. The biological observations are established in the cited studies, whereas the annotation failure mode and benchmark are proposals for testing.

Ewing sarcoma (EwS) is a stringent stress test. FET::ETS-family fusions, most commonly EWSR1::FLI1, provide a molecular anchor for malignant provenance [5,6]. Yet experimental modulation and single-cell analyses show substantial plasticity in fusion-associated programs [[7], [8], [9]]. Cells sharing fusion-anchored provenance can occupy proliferative, hypoxic, migratory, mesenchymal and matrix-producing states. If provenance evidence is weakened, a state-dominant workflow may move these cells across an annotation boundary without changing their biological origin.

## Ewing sarcoma as a mechanistic stress test

### Fusion anchors malignant provenance but not a uniform phenotype

EwS is defined by FET::ETS-family fusions [5,6]. Large genomic studies identified recurrent secondary alterations including disruption of STAG2, TP53 and CDKN2A [10,11]. This architecture is informative for atlas design: malignant provenance is comparatively well anchored, whereas phenotype remains plastic. EWSR1::FLI1 is an aberrant transcription factor and chromatin regulator that reshapes enhancer activity and directly activates or represses regulatory programs [12,13]. Direct fusion detection therefore supports malignant provenance but does not specify the regulatory configuration or phenotype of that cell.

### Fusion-anchored malignant cells occupy multiple regulatory states

Variation in EWSR1::FLI1 activity was first linked experimentally to alternative proliferative and migratory phenotypes [8]. Single-cell studies subsequently identified proliferative, oxidative-phosphorylation and hypoxic programs across defined ranges of fusion activity, as well as conserved and patient-specific malignant programs and copy-number-defined subclonal evolution [7,14] These findings define a multidimensional regulatory landscape that a binary EWSR1::FLI1-high versus EWSR1::FLI1-low model cannot capture.

Mechanistic studies explain why fusion abundance and regulatory output should remain separate. Modest and near-complete EWSR1::FLI1 depletion produced non-monotonic effects, and transient suppression followed by restoration left persistent pro-metastatic dysregulation [9]. STAG2 loss increases EWSR1::FLI1 abundance while redistributing chromatin binding and selectively amplifying microsatellite-enhancer output [15]. An autocrine TGFβ2-TGFBR2 loop sustains a hybrid configuration in which fusion-activated programs persist while mesenchymal genes, extracellular-matrix deposition and invasion are derepressed [16,17]. Fusion detection, activation, repression and phenotype are therefore distinct measurements.

### Malignant cells can converge on a stromal phenotype

CD73-positive CAF-like tumor cells provide a direct example of provenance-state decoupling. These EwS cells show a matrisome-rich program, mesenchymal features and extracellular-matrix deposition, and are enriched at tumor borders and invasive fronts. They retain EWSR1::FLI1 expression, a fusion-activated signature, proliferative capacity and tumorigenicity despite derepression of mesenchymal genes normally suppressed by the fusion [16]. They are malignant cells in a CAF-like state, not conventional stromal CAFs.

Their convergence with stroma creates a risk that reference mapping, label transfer or cluster-level marker inspection may assign ECM-rich EwS cells to a stromal compartment when provenance is not retained. We term this failure mode malignant-to-stromal annotation leakage: an annotation error, not biological conversion into bona fide stroma. Because it has not yet been systematically quantified in pan-cancer atlases, we frame it as a testable risk. The converse error is also possible: labeling every mesenchymal cell in an EwS specimen as malignant would absorb genuine fibroblasts into the tumor compartment. Orthogonal provenance and state labels allow shared programs without erasing origin (Fig. 1).

**Fig. 1:**
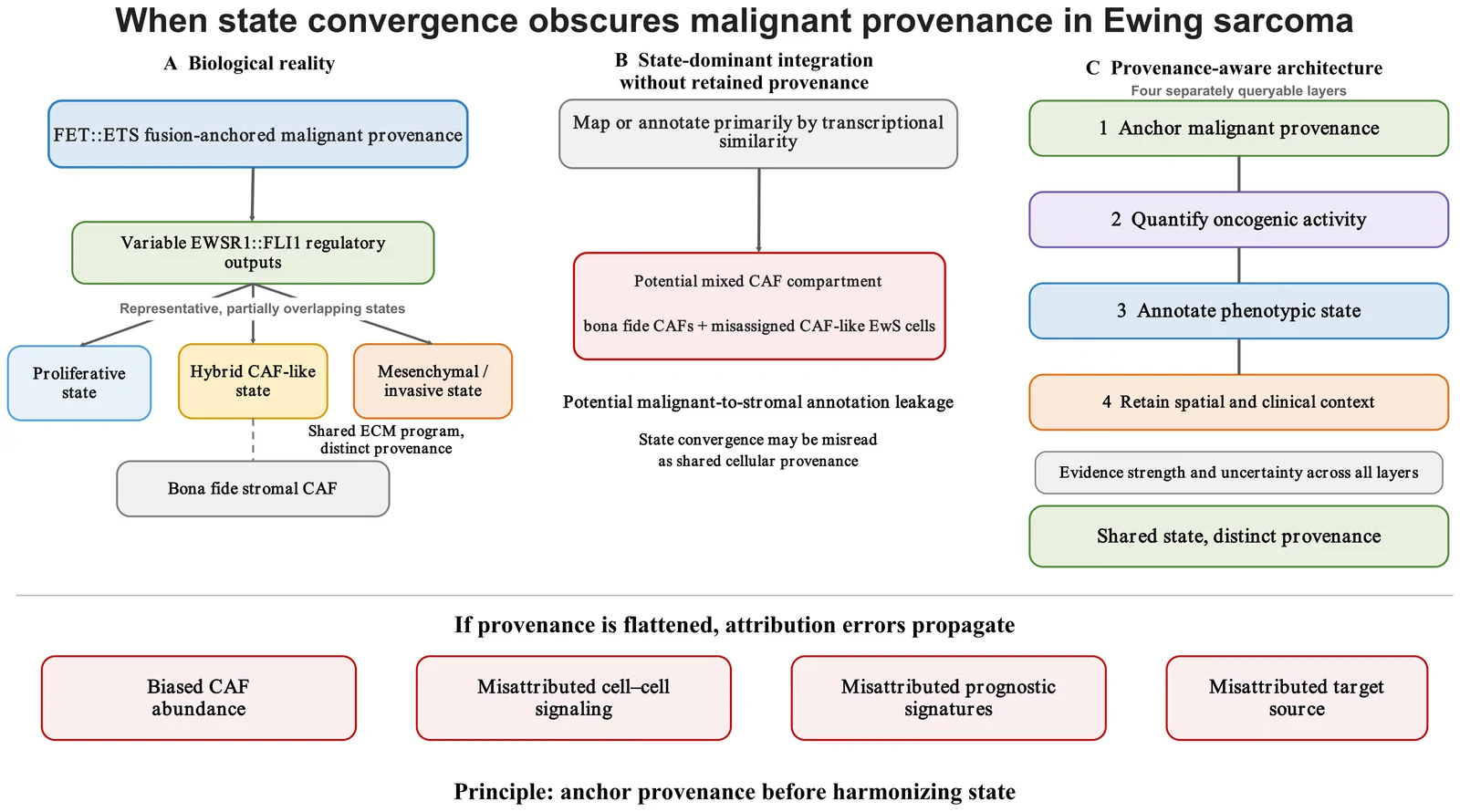
When state convergence obscures malignant provenance in Ewing sarcoma. (A) Ewing sarcoma cells sharing FET::ETS fusion-anchored malignant provenance can occupy partially overlapping proliferative, CAF-like and mesenchymal/invasive states. Variable EWSR1::FLI1 regulatory output allows malignant cells to acquire extracellular-matrix and stromal-like programs without changing their underlying provenance. Bona fide stromal CAFs may therefore share transcriptional programs with CAF-like malignant EwS cells while remaining biologically distinct. (B) When mapping or annotation is driven predominantly by transcriptional similarity and provenance evidence is not retained, CAF-like EwS cells may be assigned to a stromal compartment, creating potential malignant-to-stromal annotation leakage. This is an annotation error, not biological conversion or an inevitable consequence of integration. (C) A provenance-aware architecture keeps provenance, oncogenic activity, state and context separately queryable, with evidence strength and uncertainty retained across layers. Connecting lines do not prescribe a sequential pipeline. Provenance flattening can bias CAF abundance and misattribute signaling, prognostic signatures and target sources. Principle: anchor provenance before harmonizing state.

Supplementary Table S1 records the public data sources, sample types, reported treatment or disease status and comparators underlying these examples. The evidence spans patient specimens and experimental models, not one harmonized cohort. Goodspeed et al. profiled seven newly diagnosed, untreated primary tumors and a distinct blood/CTC component [14]. Treatment or relapse status should not be extrapolated between these components. These studies establish plasticity, not treatment- or recurrence-specific annotation-leakage frequencies.

### Why provenance errors matter for cancer inference and translation

**Biased CAF abundance.** Counting CAF-like malignant cells as stromal fibroblasts raises the estimated CAF fraction and lowers the malignant fraction. Under such misassignment, comparisons across disease stage, treatment or outcome groups may mistake a malignant matrix-producing state for increased stromal abundance.

**Misattributed cell-cell signaling.** Ligand-receptor methods operate on supplied cell labels. CellChat, for example, infers signaling between annotated groups from expressed ligands, receptors and cofactors, and benchmarking shows that both method and interaction resource materially affect the resulting networks [18,19] If an ECM-rich malignant cell is labeled as a CAF, tumor-cell-autonomous or tumor-to-tumor signaling would be reinterpreted as CAF-to-tumor signaling under the incorrect label.

**Misattributed prognostic signatures.** Single-cell references are increasingly projected into bulk cohorts to estimate cellular abundance or cell-type-specific expression [20]. A survival association assigned to stromal infiltration may instead reflect enrichment of a mesenchymal malignant state. The association may be reproducible while its cellular source is wrong, leading to different validation experiments and interventions.

**Misattributed target source.** CD73 or a matrix-remodeling program interpreted as fibroblast-derived suggests targeting supportive stroma; the same program within malignant cells suggests targeting a plastic tumor state while tracking state switching and residual clones. The stakes increase in an immunosuppressive microenvironment containing functionally impaired antigen-presenting cells [14,21]. Atlas labels sit upstream of mechanism and therapeutic prioritization.

For translational oncology, these errors become consequential when atlas-derived signatures are transferred across specimen types, disease sites or treatment contexts, or used to prioritize biomarkers and targets. Dissociated sampling can underrepresent spatially restricted cell states. Pathology-integrated spatial validation should therefore accompany high-consequence claims about stromal abundance, treatment response or target localization.

### A provenance-aware architecture for cancer single-cell atlases

The practical rule is to anchor provenance before harmonizing state. Pan-cancer integration remains compatible with this rule when four queryable layers - provenance, oncogenic activity, state and context - remain separately traceable rather than being compressed into one cell-type label. The four layers are distinct analytical dimensions, not sequential steps. Evidence tiers rank information within the malignant-provenance layer.

### Who is the cell? Anchor malignant provenance

Within the malignant-provenance layer, we propose a specificity-based evidence hierarchy. Tier 1 comprises direct clonal evidence: a patient-specific fusion transcript or breakpoint, a tumor-specific mutation with interpretable malignant provenance, a phased allele or another validated clonal genomic alteration. Tier 2 comprises independently validated entity-associated malignant transcriptional signatures, including fusion-associated programs used as supportive rather than decisive evidence. Tier 3 is supportive phenotype, such as CD99, NKX2-2 and STEAP1 in combination. Tier 4 is generic resemblance, including mesenchymal, fibroblastic, ECM, stress and cell-cycle programs. Evidence becomes less specific down the hierarchy; Tier 4 should never determine provenance alone. In the absence of direct clonal evidence, we propose that malignant assignment should generally rely on concordant evidence across multiple independent lower-tier categories, while discordant cells remain unresolved.

The hierarchy separates evidence used to identify the clone from measurements used to describe its current behavior. Entity-associated signatures can support a provenance call qualitatively, but their quantitative activity belongs in the oncogenic-activity layer. Cell-level labels should report the evidence observed, the confidence assigned and the alternatives considered. The tiers rank evidential specificity, not a universal score; their interpretation depends on tumor entity, genomic complexity, assay sensitivity and orthogonal validation.

Cell-level provenance combines a sample-level molecular diagnosis with direct or orthogonal evidence at single-cell resolution. Targeted fusion detection improves sensitivity when breakpoint-spanning reads are sparse in droplet-based single-cell RNA sequencing [22]. RNA-derived copy-number inference recovers broad aneuploidy and clonal structure, [23] with performance governed by alteration burden, data quality, reference-cell selection and algorithm [24]. EwS studies therefore combine canonical programs, fusion-associated signatures and inferred copy number [14].

### What driver program is active? Quantify oncogenic regulatory output

Fusion-detection status belongs in provenance, whereas fusion abundance and EWSR1::FLI1 activation and repression modules belong in the oncogenic-activity layer. A single high-versus-low score can conceal hybrid states in which activated targets persist while selected repressed programs are released [9,17]. Mechanistic claims should combine transcriptomic modules with chromatin, protein, perturbational or spatial measurements. In tumors without a dominant truncal driver, this layer may represent composite pathway activity rather than one genomic lesion.

### What is the cell doing? Preserve state as a separate label

Once provenance is anchored, states such as ECM-producing, hypoxic, cycling, interferon-responsive or invasive can be harmonized across patients and tumor types without merging their origins. State conservation must also be assessed at the donor level: thousands of cells from one tumor are not independent biological replicates, and cell-level differential-expression tests can inflate false discoveries [25]. Atlases should report donor contributions, use donor-aware or pseudobulk summaries and test sensitivity to influential samples.

A state reproduced across several donors is stronger evidence than a visually prominent cluster dominated by one specimen. Entity-level estimates should therefore precede cross-tumor pooling, and apparent recurrence should be re-evaluated after influential donors are removed. This safeguard addresses a different problem from provenance assignment, but both are required before an atlas state is interpreted as broadly conserved.

### Where and under what pressure? Retain spatial and clinical context

Disease site, metastatic organ, treatment exposure, viable tumor edge, necrotic interface and invasive front can alter the probability that a state is observed. The localization of CAF-like EwS cells at borders and invasive fronts illustrates why dissociated RNA alone is insufficient for consequential provenance claims [16]. Spatial transcriptomics, multiplex imaging, pathology review and matched molecular assays should be prioritized when a disputed state supports claims about invasion, immune exclusion, treatment response or targeting. Table 1 summarizes minimum and preferred safeguards. Uncertainty is a valid output, and evidence fields should remain available for audit and sensitivity analysis.

**Table 1:** Proposed safeguards for provenance-aware single-cell analysis, illustrated in Ewing sarcoma.

Domain	Illustrative Ewing sarcoma failure mode	Proposed minimum evidence	Preferred evidence for prospective studies	Consequence if ignored
Malignant provenance	CAF-like EwS cells could be assigned to the stromal compartment if malignant provenance is inferred from transcriptional resemblance.	Without direct clonal evidence, combine source-sample confirmation with concordant cell-level evidence across multiple lower-tier categories; ambiguous cells remain unresolved.	Patient-specific fusion transcript or breakpoint and/or tumor-specific clonal or subclonal genomic evidence with interpretable malignant provenance.	CAF abundance may rise; malignant fraction may fall.
Genomic anchoring	RNA-CNV-negative malignant cells may be missed.	CNA evidence interpreted jointly with fusion-associated and entity-specific transcriptional evidence.	Matched DNA/RNA profiling, allele-aware or fusion-aware evidence, and benchmarked caller sensitivity.	Malignant cells may be mislabeled; subclones lost.
Oncogenic activity	Fusion abundance or a simple EWSR1::FLI1-high/low score is treated as equivalent to regulatory output.	Separate fusion-activation and fusion-repression modules; retain fusion-detection status independently.	Fusion-aware transcriptomics combined with chromatin, protein, perturbational and/or spatial evidence.	Hybrid states may collapse; mechanism and target attribution may be distorted.
State annotation	A fusion-driven mesenchymal or ECM-producing state could be mistaken for a change in cellular provenance.	Separate provenance and state labels; score fusion-activation and fusion-repression modules independently.	Multimodal regulatory-state model incorporating fusion, chromatin, protein and spatial features.	Plasticity may be obscured; annotation leakage may result.
Replication and context	A donor-, site- or treatment-specific program is interpreted as a universal EwS state.	Donor-contribution reporting, donor-aware estimates, sampling-site and treatment metadata.	Matched primary/metastatic or pre/post-treatment sampling with external replication.	An unstable recurrent-state or clinical association may result.
Orthogonal validation	An ECM-rich malignant state could be misassigned as CAF.	Spatial/imaging evidence with provenance-linked molecular features to test malignant versus stromal assignment.	Spatial profiling combined with fusion/breakpoint or clonal validation and pathology review.	Signaling, biomarker and target sources may be misattributed.

Evidence basis: atlas integration [3]; EwS CAF-like and hybrid malignant states [16,17]; oncogenic dosage dynamics [9]; fusion detection [22]; RNA-derived CNV inference and benchmarking [23,24]; donor-level inference [25]. The four analytical layers are distinct from the provenance evidence tiers. The tiers are an entity- and assay-dependent hierarchy of evidential specificity, not a validated universal score.

Abbreviations: CAF, cancer-associated fibroblast; CNA, copy-number alteration; ECM, extracellular matrix; EwS, Ewing sarcoma.

### When should provenance flattening be expected?

We hypothesize that risk will be greatest when three conditions coexist: provenance is anchored more securely by genomic or clonal evidence than by phenotype; malignant cells can acquire programs shared with normal or stromal cells; and integration or label transfer is performed after provenance evidence has been weakened. These conditions, rather than histology alone, should define where the risk is tested. EwS meets the triad because it is fusion-defined and capable of CAF-like plasticity. Developmental reprogramming offers a related caution: in a zebrafish model, EWSR1::FLI1 expression in neural crest-derived cells drove a mesoderm-like trajectory [26]. This model does not settle the human cell of origin; it shows that current phenotype can become partly decoupled from ancestral developmental identity.

The framework is particularly relevant to tumors in which independent clonal anchors coexist with pronounced lineage plasticity or mesenchymal convergence. This separation is also relevant outside sarcoma. Spatial profiling of metastatic pancreatic cancer has demonstrated marked shifts in malignant transcriptomic lineage states across anatomical sites while independently reconstructing patient-specific clonal architecture. These findings do not establish annotation leakage; they illustrate a broader setting in which phenotypic state and clonal provenance can vary along partially independent axes [27]. Generalization should depend on demonstrating the three premises for each entity, not on listing histologies or assuming that annotation leakage has already occurred. A shared stromal-like program alone is insufficient; the relevant signal is reproducible disagreement between independent provenance and state evidence.

Fusion-defined tumors are one implementation. In glioblastoma, plastic malignant states coexist with genetic diversity, including EGFR, PDGFRA and CDK4 amplification and NF1 mutation [28]. Such genomically complex cancers require provenance assessment adapted to patient-specific copy-number alterations, somatic variants and available orthogonal genomic evidence. This example supports separating clone from state, not annotation leakage. For atlas audits, tumor entity and data quality should determine which anchors are usable; inadequate clonal support should leave provenance unresolved.

### How should provenance-flattening risk be tested?

We propose comparing state-dominant annotation, defined as cell labeling primarily from transcriptional similarity, with provenance-aware annotation in the same cells under matched preprocessing and reference conditions. Provenance metadata should be withheld only at the annotation stage in the state-dominant comparator; other inputs should be held constant. The truth set must be locked before label comparison and based on cell-level molecular evidence independent of the expression-based classifier, with spatial and pathological support where available. To prevent circular evaluation, molecular evidence supplied to provenance-aware annotation should be distinct from the prespecified, held-out evidence used for scoring, ideally obtained through orthogonal assays or non-overlapping genomic features. A sample-level EwS diagnosis alone cannot establish provenance for every cell in that specimen. In EwS, the primary endpoint is the proportion of independently fusion- or clonally anchored CAF-like malignant cells assigned to stromal labels. Reports should include sensitivity, specificity and unresolved-cell fractions with explicit denominators, using independently supported non-malignant cells to measure the converse error. Treatment-naïve, treated and recurrent specimens should be distinguished, with donor-level estimates before pooling.

Downstream analyses should then be recalculated under both annotations: CAF fraction, cellular source and direction of ligand-receptor edges, bulk deconvolution signatures, and the compartment assigned to prognostic or therapeutic targets. Robust claims should survive reasonable alternatives. A multi-entity benchmark including tumors that do and do not satisfy the three-condition rule would determine whether EwS is an instructive exception or a predictive example of a broader class. When independent cell-level molecular truth is unavailable, disagreement between annotation strategies should be reported as a sensitivity analysis rather than a validated malignant-to-stromal misclassification rate. Failure to detect reproducible misassignment would weaken the annotation-leakage hypothesis under the workflows and conditions tested, without excluding its occurrence in other biological or analytical settings.

### Boundary conditions and limitations

Three boundary conditions are important. First, mixed expression profiles require technical artifacts such as doublets and ambient RNA to be excluded before biological convergence is inferred [29,30]. Second, spatial colocalization supports but does not establish clonal provenance, while failure to detect a fusion or broad CNV in sparse transcriptomes does not prove non-malignancy. Third, the proposed architecture is not a validated classifier; its value lies in making evidence and uncertainty explicit and in generating a falsifiable comparison between state-dominant and provenance-aware annotation.

## Conclusions

Cancer single-cell atlases need common state vocabularies, but a shared vocabulary should not flatten malignant provenance. EwS shows that malignant cells can become CAF-like, matrix-producing or invasive while retaining fusion-anchored provenance, and that driver abundance is an incomplete proxy for regulatory output. An accountable atlas should keep provenance, oncogenic activity, state and context separable; expose donor contributions and uncertainty; and calibrate consequential claims to orthogonal validation. Before atlas-derived states are advanced as biomarkers, therapeutic targets or clinically meaningful microenvironmental compartments, their cellular origin should be established. The governing principle is concise: anchor provenance before harmonizing state.

## Funding

This research received no specific grant from funding agencies in the public, commercial, or not-for-profit sectors.

## Declaration of generative AI and AI-assisted technologies in the manuscript preparation process

During preparation of this manuscript, the authors used ChatGPT (OpenAI) solely for language refinement, structural organization and presentation. The authors reviewed and edited the output and take full responsibility for the content of the manuscript.

## CRediT authorship contribution statement

**Ge Zhao:** Writing – original draft, Investigation. **Zhijie Hu:** Writing – original draft, Investigation. **Baicheng Yang:** Supervision, Conceptualization. **Wei Shi:** Writing – review & editing, Supervision, Conceptualization.

## Declaration of competing interest

The authors declare that they have no known competing financial interests or personal relationships that could have appeared to influence the work reported in this article.

## Data Availability

No new datasets were generated or reanalyzed for this Opinion Article. Published data sources and sampling contexts are documented in Supplementary Table S1. The proposed annotation comparison is a future benchmark, not a completed analysis.
