# Supplementary material for: Preserving malignant provenance in single-cell cancer atlases: lessons from Ewing sarcoma plasticity
Source: Neoplasia. 2026 Nov 6;83:101392. doi: 10.1016/j.neo.2026.101392 (PMC13770630; doi:10.1016/j.neo.2026.101392)
Supplement: Supplementary file 1 [file mmc1.docx]

Supplementary Table S1 Published evidence underlying the biological examples

S1A Ewing sarcoma data sources and clinical or experimental context

This table records published data sources, not a newly analyzed cohort. Reference numbers correspond to the revised manuscript bibliography. NR means not reported at the required clinical or sample-specific resolution in the cited source; NA means not applicable. Experimental perturbation and clinical treatment status are distinguished.

| **Study and evidence type** | **Public source and assay** | **Treatment or disease context** | **Comparators and interpretation** |
| --- | --- | --- | --- |
| Aynaud et al. 2020 [7] PDX and cell-line scRNA-seq | GSE130025 SuperSeries. GSE130023: 3 PDX models, Fluidigm C1. GSE130024: 5 PDX models, 10x Chromium. GSE130019: A673/TR/shEF time course. | PDX and cultured models are not an untreated patient scRNA-seq cohort. Patient treatment or recurrence status cannot be assigned uniformly from the SuperSeries. Fusion suppression/restoration is an experimental perturbation. | Within-model EWSR1::FLI1 modulation and between-model comparisons. GSE130021 and GSE130022 provide rhabdoid and neuroblastoma control cell models, not matched normal stromal controls. |
| Aynaud et al. 2020 [7] Bone-marrow-derived MSC cultures | GSE130020, within GSE130025. The sample records describe mesenchymal stem cells isolated from EwS-patient bone marrow and cultured before Fluidigm C1 profiling; not malignant marrow cells. | Clinical pretreatment and relapse status: NR at the required patient-specific resolution. The sample protocol “No treatment” describes experimental handling and does not establish absence of prior clinical therapy. | Mesenchymal stem-cell control cultures, kept distinct from malignant PDX and EwS cell-line data. Neither a patient tumor-cell cohort nor a paired untreated-versus-relapsed clinical comparison. |
| Goodspeed et al. 2025 [14] Primary patient tumors | GSE277083. Seven primary-tumor specimens; droplet scRNA-seq and whole-genome characterization are described in the paper. Processed counts and labels are public; raw FASTQ sharing is restricted for privacy. | Seven newly diagnosed, untreated patients. The paper reports tumor site, metastases and clinical outcomes in Table 1. Later recurrence outcomes do not make the diagnostic biopsies relapsed samples. | Malignant cells versus other cell populations in the same tumors; non-malignant cells used as RNA-CNV reference. Whole-genome and available clinical cytogenetic information support genomic interpretation. |
| Goodspeed et al. 2025 [14] Blood and CTC component | GSE277083. Peripheral blood from eight patients; four also contributed primary-tumor data. CTC enrichment and scRNA-seq are distinct from tissue profiling. | Do not extrapolate the primary-cohort pretreatment status to every blood specimen. Table 1 gives clinical characteristics, but sample-time treatment and relapse status are not resolved here as a uniform blood-cohort stratum. | CTC versus blood-cell expression, and primary-tumor/CTC comparison where paired material was available. This is not a matched treatment-naïve-versus-relapsed CTC benchmark. |

Public records: [GSE130025](https://www.ncbi.nlm.nih.gov/geo/query/acc.cgi?acc=GSE130025)	[GSE130020](https://www.ncbi.nlm.nih.gov/geo/query/acc.cgi?acc=GSE130020)	[GSE130023](https://www.ncbi.nlm.nih.gov/geo/query/acc.cgi?acc=GSE130023)	[GSE130024](https://www.ncbi.nlm.nih.gov/geo/query/acc.cgi?acc=GSE130024)	[GSE277083](https://www.ncbi.nlm.nih.gov/geo/query/acc.cgi?acc=GSE277083)

Supplementary Table S1A continued

| **Study and evidence type** | **Public source and assay** | **Treatment or disease context** | **Comparators and interpretation** |
| --- | --- | --- | --- |
| Wrenn et al. 2023 [16] CAF-like malignant cells | GSE236289 SuperSeries. GSE226562: CD73-sorted bulk RNA-seq and GeoMx. GSE236284: CITE-seq of nine EwS cell lines. Patient-tissue imaging and xenograft assays are also reported. | Cell-line and xenograft treatment status is NA as a patient-therapy category. Patient material is not a uniform pretreatment scRNA-seq cohort; a treatment-stratified CAF-like-state frequency is not established. | CD73-positive versus negative malignant cells; spatial regions in experimental tumors; tissue localization. Model-derived findings must remain distinct from patient single-cell cohort evidence. |
| Wrenn et al. 2026 [17] Hybrid-state mechanism | GSE299137: RNA-seq generated for TGFβ2/hybrid-state experiments. Reused single-cell sources include GSE236284 and GSE130025; these are not independent new patient cohorts. | Experimental cytokine/pathway perturbation in EwS models, with patient-tissue observations. Uniform patient treatment or recurrence strata: NR for the mechanistic comparisons summarized here. | Matched experimental controls for pathway perturbation and state readouts. Supports TGFβ-dependent regulation, not a clinical treatment-effect estimate or atlas misassignment rate. |
| Pei et al. 2025 [27] Metastatic pancreatic cancer | GSE277783 SuperSeries; GSE274557 spatial transcriptomics and GSE277782 CosMx. Rapid-autopsy material from 13 patients, spanning primary and metastatic sites. | Five treatment-naïve and eight previously chemotherapy-treated patients are distinguished in the publication. This is pancreatic cancer, not EwS, and spatial profiling is not a dissociated EwS scRNA-seq cohort. | Within-patient primary/metastatic sites and organ-specific states; treated/untreated strata. Site and therapy histories should remain separate rather than pooled as interchangeable samples. |
| Neftel et al. 2019 Glioblastoma[28] | GSE131928: processed scRNA-seq and metadata from 28 adult/pediatric IDH-wildtype glioblastomas, profiled with Smart-seq2 and 10x. Publication also integrates genetic and functional analyses. | No treatment-naïve/relapsed comparative cohort is constructed in this Opinion. Patient-level treatment strata are not inferred from the GEO title or platform labels. | Malignant versus non-malignant populations; cell-state/genetic associations and lineage-tracing experiments in the original study. A complex-cancer example, not proof of annotation leakage. |

Public records: [GSE236289](https://www.ncbi.nlm.nih.gov/geo/query/acc.cgi?acc=GSE236289)	[GSE226562](https://www.ncbi.nlm.nih.gov/geo/query/acc.cgi?acc=GSE226562)	[GSE236284](https://www.ncbi.nlm.nih.gov/geo/query/acc.cgi?acc=GSE236284)	[GSE299137](https://www.ncbi.nlm.nih.gov/geo/query/acc.cgi?acc=GSE299137)	[GSE277783](https://www.ncbi.nlm.nih.gov/geo/query/acc.cgi?acc=GSE277783)	[GSE131928](https://www.ncbi.nlm.nih.gov/geo/query/acc.cgi?acc=GSE131928)

Supplementary Table S1B Claim to evidence mapping

The evidence establishes biological states and mechanisms. The annotation failure mode and its frequency remain prospective questions. No listed study is presented as a completed comparison of state-dominant versus provenance-aware annotation.

| **Published source** | **Conclusion supported** | **Scope and limits** |
| --- | --- | --- |
| Aynaud et al. [7] | EwS fusion-associated regulatory activity is heterogeneous, with distinct transcriptional programs across cells and experimental systems. | Marrow-derived MSC control cultures, malignant PDX and EwS cell-line components must be interpreted separately. These data do not establish the prevalence of malignant-to-stromal misassignment in reused atlases. |
| Goodspeed et al. [14] | Untreated primary EwS tumors contain conserved and patient-specific malignant programs and copy-number-defined subclonal heterogeneity. | Cohort and assay components differ. Canonical markers, fusion-associated signatures and RNA-CNV evidence support published labels but do not automatically supply classifier-independent truth for a new leakage benchmark. |
| Wrenn et al. [16] | CAF-like malignant EwS cells retain malignant properties while expressing matrix/stromal programs; experimental and spatial findings support state convergence. | Cell lines, xenografts and patient-tissue observations do not yield a treatment- or recurrence-stratified clinical frequency of this state or a measured atlas annotation-error rate. |
| Wrenn et al. [17] | TGFβ2-dependent feedback sustains a hybrid transcriptional configuration in which fusion-associated activation and mesenchymal derepression coexist. | Mechanistic pathway perturbation is not equivalent to testing atlas annotation. Reuse of earlier single-cell datasets is not independent clinical replication. |
| Pei et al. [27] | Spatially patterned pancreatic-cancer states and patient-specific clonal architecture can be analyzed separately across metastatic sites. | This is a generalization example, not evidence that pancreatic-cancer atlases have lost provenance. RNA-derived clonal inference is not automatically an independent DNA-based truth set. |
| Neftel et al. [28] | Genetic diversity, plastic malignant states and their relationships coexist in glioblastoma, illustrating adaptation beyond fusion-defined tumors. | State/genotype associations do not establish annotation leakage. Orthogonal evidence and assay-specific uncertainty remain necessary when using complex genomes for provenance assignment. |

Interpretive rule: treatment, recurrence and comparator categories apply only where the source explicitly reports them. A repository accession identifies the deposited data and does not imply that the present authors downloaded, harmonized or reanalyzed those data.

Primary-source links: [Aynaud 2020](https://pubmed.ncbi.nlm.nih.gov/32049009/)	[Goodspeed 2025](https://pmc.ncbi.nlm.nih.gov/articles/PMC12081191/)	[Wrenn 2023](https://pmc.ncbi.nlm.nih.gov/articles/PMC10801911/)	[Wrenn 2026](https://pmc.ncbi.nlm.nih.gov/articles/PMC12822632/)	[Pei 2025](https://pubmed.ncbi.nlm.nih.gov/40269162/)	[Neftel 2019](https://pubmed.ncbi.nlm.nih.gov/31327527/)
